# Genetics of Neurogenic Orthostatic Hypotension in Parkinson’s Disease, Results from a Cross-Sectional In Silico Study

**DOI:** 10.3390/brainsci13030506

**Published:** 2023-03-17

**Authors:** Guenson Chevalier, Lucas Udovin, Matilde Otero-Losada, Sofia Bordet, Francisco Capani, Sheng Luo, Christopher G. Goetz, Santiago Perez-Lloret

**Affiliations:** 1 Centro de Altos Estudios en Ciencias Humanas y de la Salud, Universidad Abierta Interamericana, Consejo Nacional de Investigaciones Científicas y Técnicas, CAECIHS, UAI-CONICET, Av. Montes de Oca 745, Buenos Aires C1147AAU, Argentina; 2 Centro de Investigaciones en Psicología y Psicopedagogía (CIPP), Facultad de Psicología y Psicopedagogía, Pontificia Universidad Católica Argentina (UCA), Av. Montes de Oca 745, Buenos Aires C1107AFB, Argentina; 3 Instituto de Ciencias Biomédicas, Facultad de Ciencias de la Salud, Universidad Autónoma de Chile, 7500912 Santiago, Chile; 4 Department of Biostatistics & Bioinformatics, Duke University, Durham, NC 27708, USA; 5 Department of Neurological Sciences, Rush University Medical Center, Chicago, IL 60612, USA; 6 Observatorio de Salud Pública, Vicerrectorado de Investigación e Innovación Académica, Pontificia Universidad Católica Argentina, Consejo Nacional de Investigaciones Científicas y Técnicas (UCA-CONICET), Av. Alicia Moreau de Justo 1300, Buenos Aires C1107AAZ, Argentina; 7 Departamento de Fisiología, Facultad de Medicina, Universidad de Buenos Aires (UBA), Buenos Aires C1121ABG, Argentina

**Keywords:** single-nucleotide polymorphism, Parkinson’s disease, neurogenic orthostatic hypotension, pathophysiology, PD-related variants

## Abstract

The genetic basis of Neurogenic Orthostatic Hypotension (NOH) in Parkinson’s disease (PD) has been inadequately explored. In a cross-sectional study, we examined the association between NOH and PD-related single-nucleotide polymorphisms (SNPs) and mapped their effects on gene expression and metabolic and signaling pathways. Patients with PD, free from pathological conditions associated with OH, and not taking OH-associated medications were included. NOH was defined as per international guidelines. Logistic regression was used to relate SNPs to NOH. Linkage-disequilibrium analysis, expression quantitative trait loci, and enrichment analysis were used to assess the effects on gene expression and metabolic/signaling pathways. We included 304 PD patients in the study, 35 of whom had NOH (11.5%). NOH was more frequent in patients with SNPs in SNCA, TMEM175, FAM47E-STBD1, CCDC62, SCN3A, MIR4696, SH3GL2, and LZTS3/DDRGK1 and less frequent in those with SNPs in ITGA8, IP6K2, SIPA1L2, NDUFAF2. These SNPs affected gene expression associated with the significant hierarchical central structures of the autonomic nervous system. They influenced several metabolic/signaling pathways, most notably IP3/Ca++ signaling, the PKA-CREB pathway, and the metabolism of fatty acids. These findings provide new insights into the pathophysiology of NOH in PD and may provide targets for future therapies.

## 1. Introduction

Parkinson’s is the second most frequent neurodegenerative disorder worldwide [1]. Patients are affected by motor and non-motor symptoms, with the latter being the most disturbing [2]. Pathogenically, PD likely encompasses many genetic–molecular entities resulting in lesions to different structures within the central or peripheral nervous system. The deposition of alpha-synuclein in the cellular soma, leading to the formation of Lewy bodies, appears to be one of the main events leading to neurodegeneration [2]. Other factors contributing to the neurodegenerative process include mitochondrial dysfunction, synaptic alterations, the disruption of calcium homeostasis, and neuroinflammation [2].

Familial PD accounts for about 5% of all patients and has been connected with rare mutations [3,4]. Recent evidence indicates that genetic mutations also contribute in non-negligible ways to sporadic PD [5]. A recent meta-analysis of genome-wide association studies (GWAS) included the analysis of 7.8M SNPs in 37.7K cases, 18.6K UK Biobank proxy-cases (having a first-degree relative with PD), and 1.4M controls [5]. The authors could identify 90 variants that explained 16–36% of the heritable risk of PD depending on prevalence. Interestingly, the presence of some mutations determines distinct phenotypes. For example, in PD patients with mutations in the Leucine-rich repeat kinase 2 (LRRK2) gene, the disease progresses slower than those without mutations and are less frequently affected by non-motor symptoms, including olfaction, REM-sleep behavior disorders, and cognitive dysfunction [6]. PD may also relate to mutations in the GBA gene, encoding the lysosomal enzyme glucocerebrosidase (Gcase) [5]. A recent meta-analysis has shown that PD patients with *GBA* mutations suffer from accelerated progression of the disease, with more frequent motor fluctuations, depression, and dementia compared to non-affected patients [7].

Neurogenic Orthostatic Hypotension (NOH) is a common and disabling non-motor feature affecting over 30% of Parkinson’s disease (PD) patients [8,9], sometimes observed in recently diagnosed patients even before starting anti-Parkinsonian treatment [10]. NOH can be defined as a sustained reduction in systolic and/or diastolic blood pressure (BP) after orthostasis or a head-up tilt test [11]. In the worst cases, symptoms include generalized weakness, lightheadedness, dizziness, or even syncope [8,11,12]. Orthostatic hypotension can result from aging, environmental conditions, comorbidities, or drug treatments [12,13,14]. However, in PD, hypotension results from the neurodegenerative process itself, thus representing a case of Neurogenic Orthostatic Hypotension (NOH). NOH causes substantial morbidity and mortality, mainly from falls’ sequelae, and is an independent heart disease morbidity and mortality, stroke, and cardiovascular and all-cause mortality risk factor [15,16]. Upon cerebral blood flow autoregulatory mechanism compromise, hypoperfusion episodes lead to white matter lesions, cognitive disturbance, and dementia [17,18,19,20].

Sympathetic dysfunction is a hallmark of NOH in PD. Compared with age-matched controls, PD patients have low baroreflex-cardiovagal gain, which is worse in NOH patients [8,21]. The adrenergic response to orthostasis, i.e., increased norepinephrine release in blood vessels’ sympathetic terminals, is reduced, and these patients’ circulating norepinephrine levels are decreased [8,21]. The deposition of Lewy bodies in the locus coeruleus and sympathetic postganglionic cells’ loss has been implicated [8,21]. Heart sympathetic denervation is a hallmark of NOH in PD [8,21].

The genetic basis of NOH in PD has yet to be sufficiently explored. Specific single-nucleotide polymorphisms (SNPs) related to PD have been shown to affect the autonomic nervous system and the risk of developing NOH [22,23]. Indeed, carriers of LRRK2 mutations showed reduced autonomic impairments compared with non-carriers [23]. PD patients carrying the chromosomal triplication of SNCA may display evidence of sympathetic cardiac denervation and are frequently associated falls up to 3 years before disease onset [22,23]. Likewise, mutations in the PARK2 may also result in sympathetic cardiac denervation [22].

We explored the association between PD-related SNPs and NOH to test the hypothesis that other PD-related SNPs may also be associated with NOH. We mapped the effects of these SNPs on gene expression and metabolic or signaling pathways.

## 2. Materials and Methods

### 2.1. Participants

The Parkinson’s Progression Markers Initiative (PPMI) is an ongoing multicenter observational study identifying disease biomarkers in PD patients attending clinical centers from America to Oceania [24]. All participants signed written informed consent, and the review board approved the protocol of each center. Information was de-identified and shared with involved and uninvolved investigators. We only extracted information from each participant’s baseline visit.

We excluded patients with pathological conditions and/or medications associated with orthostatic syndromes, including diabetes, alcoholism, polyneuropathy, and amyloidosis, and users of calcium channel blockers, diuretics, angiotensin-converting enzyme inhibitors, AT-1 receptor blockers, nitrates, α-adrenoreceptor antagonists, opioids, or tricyclic antidepressants. In addition, patients with missing data or those in whom the PD diagnosis was changed during the first five years after the diagnosis of PD were excluded. Recent data indicate that five years of follow-up is enough to reach a clinical diagnosis of PD or Parkinsonism in more than 95% of patients, thus minimizing the possibility of false diagnosis [25].

Blood pressure measurement and patients’ NOH classification

As part of the baseline evaluation, blood pressure was measured 5–10 min after resting supine and 1–3 min after standing up [24]. NOH was defined as a fall in systolic BP of ≥20 mm Hg (or ≥30 mm Hg in hypertensive patients) and/or a fall in diastolic BP of ≥10 mm Hg within 1–3 min when standing upright [11].

### 2.2. Genomic Data Processing

As part of the screening or baseline visit, blood was drawn, and whole-genome sequencing was performed using a Macrogen Inc. (Seoul, South Korea) sequencer on whole-blood-extracted DNA samples [24]. DNA samples (1 µg) were fragmented with the Covaris System (Covaris, Woburn, MA, USA), and prepared following the Illumina TruSeq DNA sample preparation guide (Illumina Inc, San Diego, CA, USA) to obtain a final 300–400 bp average insert size library. Libraries were multiplexed and sequenced on the Illumina HiSeq X platform (Illumina Inc, San Diego, CA, USA). Paired-end read sequences were initially aligned to the GRCh37-hs37d5 genome using the Burrows–Wheeler aligner maximal exact matches algorithm (BWA-MEM v0.7.13). The Bamsormadup2 tool (v2.0.87) filtered duplicates and sorted aligned bam files. After filtering duplicated read sequences, the reads were realigned and recalibrated using the GATK pipeline (v3.5). A haplotype caller in the GATK pipeline was used to call variants, including single-nucleotide variants (SNVs) and small In/Dels, and generate genome VCFs. Using the hg38 aligned cohort VCF files from the whole-genome sequencing data, genotype information was extracted using BCF tools and PLINK [26]. We considered the alleles of the 72 variants available in the PPMI database associated with an increased risk of PD as identified in a recent large case–control study [5]. We focused on SNPs with a minimum call rate of 95%, a minor allele frequency (MAF) > 1%, and Hardy–Weinberg equilibrium *p*-values > 0.05.

### 2.3. Linkage Disequilibrium Analysis

We performed a linkage disequilibrium (LD) analysis to identify all the SNPs co-segregated with the PD-related SNPs associated with NOH. The analysis was performed using the lDlink Proxy application of the lDlink platform of the National Institute of Cancer (USA) [27]. We used R-squared of ≥ 0.5, D’ ≥ 0.8, and MAF > 0.01 as parameters for the analysis.

### 2.4. Expression Quantitative Trait Loci (eQTL) Analysis

We studied the effects of PD-related SNPs associated with NOH on the expression of other genes by performing an eQTL analysis using the lDlink platform [27]. Genes showing an R-square higher than 0.5 and a D’ higher than 0.8 were considered related. American, European, and Latino populations were examined in this study for being the most representative of the PPMI database.

### 2.5. Enrichment Analysis of SNPs Associated with NOH

The PD-related SNPs associated with NOH and other SNPs in LD with the former ones were the substrate of the enrichment analysis. This analysis allowed us to explore the metabolic or signaling pathway in which the genes containing the SNPs associated with NOH are involved. For this analysis, we used the SNPnexus software (Barts Cancer Institute, London, UK) [28]. We considered paths with a *p*-value < 0.05 as enriched.

### 2.6. Statistical Analysis

Numerical variables were expressed as means ± standard deviation and the categorical ones in percentages. Differences between PD patients with and without NOH were analyzed with a T-test or Chi-square test.

We used a logistic regression model to identify PD-related SNPs independently associated with NOH, adjusting for sex, age, and anti-Parkinsonian treatments (KNIME platform 4.1.0, Knime AG, Zurich, Switzerland). All SNPs with *p*-values < 0.05 were selected for annotation, LD, enrichment, and eQTL analyses, as already described. The Benjamini and Hochberg step-up procedure was used to control the False Discovery Rate and adjust the *p*-values accordingly.

## 3. Results

For this study, we selected 304 patients. We observed NOH in 35 of the 304 participants (11.5%). Table 1 shows the characteristics of patients with or without NOH. A mildly higher male prevalence in patients with NOH was the only significant difference between the two groups.

### 3.1. Association between SNPs in PD-Related Genes and NOH

Logistic regression analysis showed that 13 of the 72 PD-related SNPs were independently related to NOH (Table 2). The annotation of these SNPs is shown in Table 3. According to an additive model, NOH was more frequent in patients with risk genotypes in SNCA (two SNPs), TMEM175, FAM47E-STBD1, CCDC62, SCN3A, MIR4696, SH3GL2, and LZTS3/DDRGK1. Conversely, according to our additive model, risk genotypes in ITGA8, IP6K2, SIPA1L2, and NDUFAF2 reduced the NOH risk. Age was the only covariate significantly associated with NOH (OR, 95% CI = 1.04, 1.01-1.07; *p* = 0.003).

All the SNPs associated with NOH entered LD analysis. No data were available for the SNP rs104893877. After quality control, we obtained 3628 SNPs in strong LD with the NOH-associated SNPs (Table 4).

### 3.2. eQTL Gene Expression Mapping Analysis

**Figure 1:**
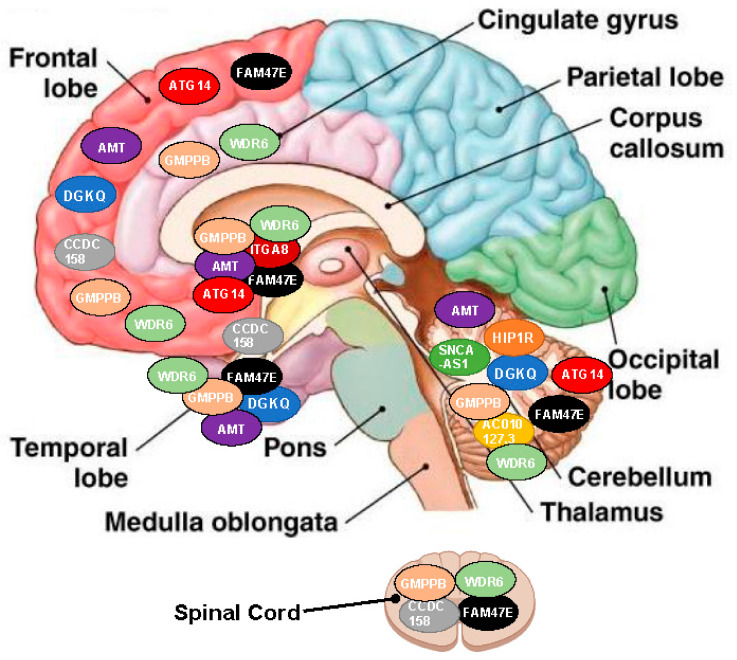
Map of the genes influenced by the SNPs (based on linkage disequilibrium analysis and eQTL).

We found that some NOH-associated SNPs altered the expression of genes such as DGKQ, HIP1R, SNCA-AS1, AC010127.3, CCDC 158, FAM47E, ITGA8, AMT, GMPPB, WDR6, and ATG14, as shown in Table 5. These are expressed in the hippocampus, frontal cortex, anterior cingulate cortex, amygdala, cerebellum, spinal cord, basal ganglia (caudate, accumbens, and putamen nuclei), and hypothalamus (Figure 1).

### 3.3. Enrichment Analysis of SNPs Associated with NOH

The cellular functions associated with the NOH-associated SNPs and other SNPs in LD with the former ones are presented in Table 6. These SNPs participated in neural growth and migration, IP3 signaling pathway, lysosome/vesicle trafficking, signaling by non-receptor tyrosine kinases, L1/Ankirins interaction, elastic fiber formation, IFN-alpha/beta signaling, mitochondrial functioning, amyloid fiber formation, PKA-CREB pathway, fatty acid, carnitine metabolism, and tRNA aminoacylation.

## 4. Discussion

The genetic basis of NOH in PD has yet to be extensively studied. In our cross-sectional study, 13 SNPs out of 72 examined variants were independently associated with NOH. Although our findings do not answer questions about the genetic impact on disease progression, they are compelling in patients with NOH, in whom genetic markers associated with dysautonomia can be identified. We mapped the expected consequences of these SNPs on gene expression and the function of the metabolic/signaling pathways because recent data suggest that PD likely encompasses a variety of genetic–molecular entities, resulting in a hard-to-predict, broad, and heterogeneous spectrum of non-motor symptoms [2]. Our results explicitly related to NOH suggest that the non-motor symptom profile that an individual PD patient manifests may partly depend on the individual’s genetic background. If our findings are replicated for other non-motor baseline symptoms, unique phenotypic patterns typically considered part of the primary neurodegenerative process of PD or its treatment may be more accurately linked to the implicit genetic makeup of individuals. This gene-based concept of risk factors for non-motor manifestations within the more confined homogeneity of nigrostriatal degeneration of PD may help us to understand the vast heterogeneity of phenotypes at disease onset and the different patterns of clinical progression.

Prior gene studies have reported that SNPs in LRRK2 reduce [23], while mutations in SNCA and PARK2 increase [22,23] autonomic impairment risk. In our study, SNP rs34637584 in LRRK2 reduced (OR = 0.16), while the SNPs rs3910105 and rs104893877 in SNCA increased NOH risk (OR = 3.05 and 55.29, respectively). Interestingly, SNPs in SNCA have also been associated with autonomic failure in other alpha-synucleinopathies such as Multiple System Atrophy [22]. In our study, NOH was also related to SNPs in other genes, suggesting a more substantial genetic influence than so far considered. Our statistical analysis was explicitly designed to rule out confounding effects such as age, sex, and any medications associated with autonomic impact. We explored the effects of the genes connected with NOH on the expression of other genes and metabolic/signaling pathways function using eQTL and Enrichment analyses (Table 5 and Table 6). Post-ganglionic sympathetic denervation is considered the main event in the pathophysiology of autonomic dysfunction [29]. However, specific PD-related factors leading to sympathetic denervation and other systems’ alterations have not been satisfactorily studied. There is evidence of the importance of the associations we observed between NOH-related SNPs and other genes. For example, the SCN3A increased AC010127.3 expression, which encodes the SCN1A and SCN9A antisense RNA1 (SCN1A-AS1) in the cerebellum. Carriers of SCN9A mutations are frequently affected by autonomic dysfunction [30]. SNCA increased MMRN1 gene expression, associated with late-onset autonomic dysfunction and Parkinsonism after duplication in the Swedish proband [31]. We found these genes were expressed in the hippocampus, frontal cortex, anterior cingulate cortex, amygdala, cerebellum, spinal cord, basal ganglia (caudate, accumbens, and putamen nuclei), and the hypothalamus, all brain regions involved in the autonomic nervous systems’ activation and regulation [32]. As mentioned, NOH in PD has been traditionally considered as resulting mainly from postganglionic sympathetic loss [29]. Our results challenge this notion, suggesting a more prominent dysfunction involving the peripheral and central nervous systems.

Previous evidence has stressed the influence of specific cellular functions related to genes connected with NOH (Table 4). The inositol phosphate metabolism, linked to IP6K2, has been associated with autonomic Ca ++ oscillations’ regulation in cardiac cells [33]. Protein-kinase A and CREB (cAMP response element-binding protein) are involved in adrenergic receptors’ signaling pathways [34]. ELOVL7 participates in elongating fatty acids [35], which have been implicated in blood pressure control. A recent trial showed that long-chain Omega-3 polyunsaturated fatty acid supplementation reduced blood pressure fall after undergoing graded lower-body negative pressure [36]. Omega-3 fatty acid supplementation did not change muscle sympathetic nerve activity at rest but augmented sympathetic outflow in response to physiological stressors such as the ischemic handgrip or cold pressor test [37]. IP6K2 is transduced into the enzyme inositol hexakisphosphate kinases (IP6K), essential for phosphate regulation in vivo [38]. IP6K hyperactivity is involved in hyperphosphatemia, and chronic IP6K inhibition improved kidney function in a rat model of chronic kidney disease [38]. Interestingly, a high circulating phosphate concentration, which may reflect vascular calcification, was associated with isolated diastolic OH in a recent study [39]. The involvement of phosphate metabolism in OH in PD has yet to be studied. The SCN2A gene encodes a sodium channel that modifies neurons’ excitability [40]. SCN2A-related disorders, which manifest with epilepsy, autism, intellectual disability, or ataxia and chorea, may also involve dysautonomia [41]. A meta-analysis of 74 microarray experiments available on public databases in spontaneously hypertensive and Lyon hypertensive rats showed that SLC25A20 was more frequently expressed in the hypertensive rats than the wild controls [42]. SLC25A20 encodes a Mitochondrial Carnitine Acyl-carnitine Carrier that participates in the energy metabolism in cardiovascular tissues [43]. We acknowledge that other pathways connected with NOH are more challenging to explain and warrant further studies (see Table 4).

Our study presents some limitations. We only used data from the baseline visit and anchored our classification on a single set of orthostatic blood pressure assessments. Furthermore, given the outpatient office-based recruitment methods, NOH was defined based on blood pressure changes after standing up rather than on a traditional tilt table test [4]. Due to the small sample size, we may have missed some SNPs associated with NOH out of reduced power. Our logistic regression model may have suffered from some degree of overfitting. We did not explore the effects of other forms of dysautonomia or conditions commonly related to cardiovascular dysautonomia, such as REM Sleep Behavior Disorder. Finally, we exclusively focused on NOH associations with PD-related SNPs, leaving out other potentially related genes such as LMNB1, CHRNA3, GBE1, and DBH [44,45,46,47]. Work for the future will be to use the identified markers to examine the 304 subjects longitudinally to detect if gene markers portend a high risk of NOH development over time or in response to dopaminergic treatment when introduced. As indicated, our model offers the same opportunity to study genetic underpinnings for other non-motor attributes of PD, namely apathy, anxiety, and depression, all readily available components of Part I of the MDS-UPDRS, the standard PD rating scale used in the PPMI study.

## 5. Conclusions

Having mapped the effects of 13 SNPs associated with NOH on the expression of other genes and on metabolic and signaling pathways, we offer these markers as potential new therapeutic targets for PD-associated NOH. Most importantly, our analysis supports the involvement of central nervous structures rather than only a postganglionic dysfunction in the pathogenesis of NOH in PD, challenging long-standing traditional views. Currently available drugs for NOH management increase adrenergic vascular tone or renal fluid retention with secondary adverse effect risks, including supine hypertension. New treatments are thus needed. We identified some targets for treating NOH, including fatty acids, IP6K inhibition, and the Mitochondrial Carnitine Acyl-carnitine Carrier. More research is required to validate these targets.

## Figures and Tables

**Table 1:** Characteristics of PD patients with and without Neurogenic Orthostatic Hypotension.

	NOH (*n* = 35)	Without NOH (*n* = 269)	*p*-Value
Age (years)	61.10 ± 9.7	58.40 ± 9.90	0.122
Males	21 (60.00%)	157 (58.15%)	0.034
Age at PD onset (years)	60.40 ± 9.95	57.45 ± 9.89	0.106
PD duration (months) *	7 (1–64)	5 (1–84)	0.276
Hoehn and Yahr	1.67 ± 0.53	1.63 ± 0.53	0.616
MDS-UPDRS Total score	34.14 ± 20.39	32.76 ± 16.20	0.706
Part I score	6.05 ± 4.99	6.14 ± 4.45	0.876
Part II score	6.52 ± 5.51	6.33 ± 4.96	0.840
Part III score	21.15 ± 12.23	19.77 ± 10.16	0.530
Part IV score	2.03 ± 3.21	1.47 ± 2.61	0.660
Anti-Parkinsonian drugs	8 (23.53%)	77 (28.52%)	0.779
L-DOPA	5 (14.7%)	52 (19.26%)	0.881
Dopamine Agonist	3 (8.82%)	41(15.18%)	0.359
Other drugs	4(11.76%)	56 (20.74%)	0.523
Levodopa-equivalent daily dose (mg/day)	920 ± 499	620 ± 828	0.169
CSF Biomarkers			
tTau	172.3 ± 52.91	165.56 ± 59.73	0.500
pTau	14.45 ± 5.35	13.95 ± 5.22	0.640
a-Synuclein	95.595 ± 32.5	104.71 ± 43.9	0.390
Neurofilament Light Chain (NFLC)	120.13 ± 68.67	100.24 ± 60.8	0.290

* Median (Min, Max).

**Table 2:** SNPs associated with Neurogenic Orthostatic Hypotension in PD by logistic regression analysis.

SNP (Gene)	Whole Sample (*n* = 304)	NOH (*n* = 35)	Without NOH (*n* = 269)	OR (95% CI)	*p*-Value	Adjusted *p*-Value
rs104893877 (SNCA)	CC:292 (96.10%) CT:12 (3.90%) TT:0	CC:31 (88.57%) CT:4 (11.43%) TT:0	CC:261 (97.03%) CT:8 (2.97%) TT:0	58.03 (2.95–190)	0.012	0.031
rs34311866 (TMEM175)	TT:180 (59.21%) TC:105 (34.54%) CC:19 (6.25%)	TT:17 (48.57%) TC:13 (37.14%) CC:5 (14.29%)	TT:163 (60.59%) TC:92 (34.20%) CC:14 (5.21%)	8.46 (2.98–29.81)	<0.001	0.002
rs6812193 (FAM47E- STBD1)	CC:132 (43.42%) CT:133 (43.75%) TT:39 (12.83%)	CC:14 (40.00%) CT:15 (42.86%) TT:6 (17.14%)	CC:118 (43.87%) CT:118 (43.87%) TT: 33 (12.27%)	3.67 (1.44–11.00)	0.010	0.031
rs11060180 (CCDC62)	AA:101 (33.22%) AG:141 (46.38%) GG:62 (20.39%)	AA:8 (22.86%) AG:16 (45.71%) GG:11 (31.43%)	AA:93 (34.57%) AG:125 (46.46%) GG:51 (18.95%)	3.54 (1.53–9.67)	0.007	0.031
rs353116 (SCN3A)	CC:119 (39.15%) CT:140 (46.10%) TT:45 (14.80%)	CC:12 (34.28%) CT:14 (40.00%) TT:9 (25.71%)	CC:107 (39.77%) CT:126 (46.84%) TT:36 (13.38%)	3.48 (1.39–10.10)	0.012	0.031
rs3910105 (SNCA)	AA:101 (33.22%) AG:146 (48.03%) GG:57 (18.75%)	AA:5 (14.28%) AG:22 (62.86%) GG:8 (22.86%)	AA:96 (35.68%) AG:124 (46.1%) GG:49 (18.22%)	3.48 (1.31–10.46)	0.017	0.034
rs329648 (MIR4696)	CC:113 (37.17%) TC:148 (48.68%) TT:43 (14.14%)	CC:8 (22.86%) TC:24 (68.57%) TT:3 (8.57%)	CC:105 (39.03%) TC:124 (46.09%) TT:40 (14.87%)	3.47 (1.27–11.54)	0.023	0.034
rs13294100 (SH3GL2)	GG:137 (45.07%) GT:137 (45.07%) TT:30 (9.86%)	GG:11 (31.43%) GT:20 (57.14%) TT:4 (11.43%)	GG:126 (46.84%) GT:117 (43.49%) TT:26 (9.66%)	3.34 (1.27–10.27)	0.021	0.034
rs55785911 (LZTS3/DDRGK1)	GG:117 (38.48%) GA:143 (47.04%) AA:44 (14.47%)	GG:10 (28.57%) GA:18 (51.42%) AA:7 (20.00%)	GG:107 (39.41%) GA:125 (46.47%) AA:37 (13.75%)	2.52 (1.03–6.67)	0.048	0.048
rs10906923 (ITGA8)	AA:140 (46.1%) AC:124 (40.79%) CC:40 (13.16%)	AA:18 (51.43%) AC:14 (40.03%) CC:3 (8.57%)	AA:122 (45.35%) AC:110 (40.89%) CC:37 (13.75%)	0.37 (0.13–0.91)	0.042	0.048
rs12497850 (IP6K2)	TT:138 (45.39%) TG:140 (46.05%) GG:26 (8.55%)	TT:16 (45.71%) TG:19 (54.28%) GG: 0	TT:122 (45.35%) TG:121 (44.98%) GG:26 (9.66%)	0.29 (0.09–0.80)	0.024	0.035
rs10797576 (SIPA1L2)	CC:206 (67.76%) CT:91 (29.93%) TT:7 (2.30%)	CC: 25 (71.43%) CT:10 (28.57%) TT: 0	CC:181 (67.28%) CT:81 (30.11%) TT:7 (2.61%)	0.19 (0.04–0.74)	0.027	0.035
rs2694528 (NDUFAF2)	CC:242 (79.60%) CA:57 (18.75%) AA:5 (1.64%)	CC:30 (85.71%) CA:5 (14.28%)	CC:212 (78.81%) CA:52 (19.33%) AA:5 (1.86%)	0.13 (0.01–0.88)	0.048	0.048

Age, sex, and treatment with anti-Parkinsonian drugs were introduced as covariates in the logistic model. OR (95% CI) = Odds Ratio (95% Confidence Interval). An additive model was assumed. *p*-values were adjusted by the Benjamini and Hochberg step-up procedure.

**Table 3:** Annotation of SNPs associated with NOH.

SNP	Chr	Position (bp)	Ref	ALT	MAF SNPnexus	MAF in the Study	Gene	Gene Position	HWE
rs10797576	1	232,664,611	C	T	0.127396	0.1728	SIPA1L2	Intronic	0.802
rs10906923	10	15,569,598	C	A	0.460863	0.3389	ITGA8	Intronic	0.194
rs329648	11	133,765,367	T	T	0.464457	0.3854	MIR4696	Intronic	0.746
rs34637584	12	40,734,202	G	A	0.000400	0.1096	LRRK2	3-UTR. coding nonsyn	0.489
rs11060180	12	123,303,586	A	G	0.251597	0.4369	CCDC62	Intronic	0.327
rs11158026	14	55,348,869	C	C	0.489816	0.3317	GCH1	Non-coding. Intronic	0.496
rs353116	2	166,133,632	C	T	0.442692	0.3787	SCN2A	Non-coding. Intronic	0.9105
rs55785911	20	3,153,503	G	A	0.39976	0.3833	LZTS3	Intronic	0.937
rs12497850	3	48,748,989	G	G	0.26857	0.3173	IP6K2	Non-coding. Intronic	0.298
rs34311866	4	951,947	T	C	0.139976	0.2342	TMEM175	3-UTR	0.503
rs6812193	4	77,198,986	C	T	0.313299	0.3422	FAM47E-STBD1	Intronic	0.725
rs3910105	4	90,682,571	A	G	0.307708	0.4302	SNCA	Intronic	0.829
rs104893877	4	90,749,300	C	T	-	0.02	SNCA	Coding non syn	0.270
rs2694528	5	60,273,923	C	C	0.134585	0.108	NDUFAF2	Intronic	0.949
rs13294100	9	17,579,690	T	G	0.457268	0.322	SH3GL2	Non-coding. Intronic	0.621

bp= base pair. Chr: chromosome where an SNP is located. Ref: allele from the reference genome. ALT: allele of the participants in the study is called an alternative allele. MAF: minor allele frequency. HWE: Hardy–Weinberg equilibrium.

**Table 4:** Results of the linkage-disequilibrium-based gene expression analysis.

SNP (Gene)	SNP in LD	Chr	Influence on Gene	Gene Expressed	Effect Size	*p*-Values	R^2^	D´
rs34311866 (TMEM175)	27	4	DGKQ	BHPC, BCRB, BFC	−0.47/ −0.19	5.90 × 10^−7^	0.65/1.00	0.86/1.00
rs34311866 (TMEM175)	1	12	HIP1R	BCRB	0.22	3.90 × 10^−6^	1.00	1.00
Rs3910105 (SNCA)	61	4	SNCA-AS1	BCRB	−0.63/ −0.39	1.66 × 10^−12^/ 2.11 × 10^−5^	0.62/1.00	0.86/1.00
Rs353116 (SCN2A)	2	2	AC010127.3	BCRB	0.59	9.00 × 10^−6^	0.17	0.88
Rs6812193 (FAM47E-STBD)	14	4	CCDC158	BC, BFC, BSC	0.32/0.48	1.20 × 10^−5^	0.5/1.00	0.97/1.00
Rs6812193 (FAM47E-STBD)	99	4	FAM47E	BCB, BCRB, BC, BFC, BHPC, BNA, PB, BSC	0.26/0.45	7.59 × 10^−5^/ 8.78 × 10^−9^	0.91/1.00	0.99/1.00
Rs10906923 (FAM47E-STBD)	200	10	ITGA8	BCB, BC, BHY, BNA	−0.41/ −0.31	4.49 × 10^−5^/ 7.67 × 10^−8^	0.53/ 1.00	0.84/ 1.00
Rs12497850 (IP6K2)	1232	3	AMT	BCRB, BC, BCB, BNA, BFC, BHPC, BP	0.19/ 0.67	2.02 × 10^−4^/ 5.28 × 10^−27^	0.57/ 1.00	0.82/ 1.00
Rs12497850 (IP6K2)	351	3	GMPPB	BAMY, BAC, BCRB, BC, BFC, BHPC, BP, BSC, BSN	−0.51/ −0.51	1.48 × 10^−10^/ 4.20 × 10^−4^	0.57/ 0.88	0.82/0.95
Rs12497850 (IP6K2)	1602	3	WDR6	BAMY, BCRB, BCB, BC, BFC, BHPC, BNA, BP, BSC, BSN	−0.45/ 0.40	2.03 × 10^−27^/ 1.89 × 10^−4^	0.50/ 1.00	0.88/ 1.00
Rs11158026 (GCH1)	39	14	ATG14	BFC, BP, BCRB	−0.43/ 0.35	1.62 × 10^−8^/ 3.20 × 10^−5^	0.50/ 1.00	0.82/1.00

Chr: chromosome in which the SNPs in LD could be found. BHPC: Brain—hippocampus. BCRB: Brain—cerebellum. BFC: Brain—frontal cortex. BC: Brain—cortex. BSC: Brain—spinal cord. BCB: Brain—caudate (basal ganglia). BNA: Brain—nucleus accumbens (basal ganglia). BP: Brain—putamen (basal ganglia). BHY: Brain—hypothalamus. BAMY: Brain—amygdala. BAC: Brain—anterior cingulate cortex. BSN: Brain—anterior cingulate cortex. Minimal and maximal values are shown for effect size, *p*-value, R^2^, and D’ parameters. The most relevant SNPs found in LD are presented in this table.

**Table 5:** Gene expression mapping based on linkage disequilibrium analysis and eQTL.

NOH-Associated SNP	Targeted Genes	Localization and Effect Direction (Arrow)									
		Hippocampus	Frontal Cortex	Anterior Cingulate Cortex	Amygdala	Cerebellum	Spinal cord	Caudate	Accumbens	Putamen	Hypothalamus
rs34311866	DGKQ	↓	↓	-	-	↓	-	-	-	-	-
	HIP1R	-	-	-	-	↑	-	-	-	-	-
rs3910105	SNCA-AS1	-	-	-	-	↓	-	-	-	-	-
rs353116	AC010127.3	-	-	-	-	↑	-	-	-	-	-
rs6812193	CCDC 158	-	↑	-	-	-	↑	-	-	-	↑
	FAM47E	↑	↑	-	-	↑	↑	↑	↑	↑	-
rs10906923	ITGA8	-	-	-	-	-	-	↓	↓	-	-
rs12497850	AMT	↑	↑	-	-	↑	-	↑	↑	↑	-
	GMPPB	↓	↓	↓	↓	↓	↓	-	-	↓	-
	WDR6	↓/↑ *	↓/↑ *	↓/↑ *	-	↓/↑ *	↓/↑ *	↓/↑ *	↓/↑ *	↓/↑ *	-
rs11158026	ATG14	-	↓/↑ *	-	-	↓/↑ *	-	-	-	↓/↑ *	-

* Depending on the genes in LD with NOH-related SNPs.

**Table 6:** Enrichment analysis of SNPs associated with NOH.

Genes Involved	Metabolic Pathway	*p*-Value	Adjusted *p*-Values
IP6K2	Synthesis of inositol phosphate in the nucleus	0.003	0.047
	Inositol phosphate metabolism	0.039	0.047
SH3GL2	Retrograde neurotrophin signaling	0.012	0.047
	InlB-mediated entry of listeria monocytogenes into a cell	0.013	0.047
	Listeria monocytogenes entry into host cells	0.017	0.047
	Negative regulation of MET activity	0.017	0.047
	EGFR downregulation	0.026	0.047
	Signaling by EGFR	0.042	0.047
	Lysosome vesicle biogenesis	0.029	0.047
	Recycling pathway of L1	0.040	0.047
	Golgi-associated vesicle biogenesis	0.046	0.047
SCN2A	Interaction between L1 and ankyrins	0.026	0.047
	Sodium channel	0.035	0.047
SH3GL2-SCN2A	L1CAM interactions	0.004	0.047
ITGA8	Molecules associated with elastic fibers	0.032	0.047
	Elastic fiber formation	0.037	0.047
NDUFAF2	Mitochondrial complex I biogenesis	0.041	0.047
PRKAR2A	ROBO receptors bind AKAP5	0.013	0.047
	AC-mediated CREB1 phosphorylation	0.018	0.047
	PKA activation in glucagon signaling	0.025	0.047
	PKA activation	0.027	0.047
	PKA-mediated phosphorylation of CREB	0.029	0.047
	DARPP-32 events	0.035	0.047
	Glucagon signaling in metabolic regulation	0.048	0.048
SLC25A20	Carnitine metabolism	0.019	0.047
ELOVL7	Synthesis of very-long-chain fatty acyl-CoA	0.035	0.047
	Fatty acid metabolism	0.028	0.047
QARS	Mitochondrial tRNA aminoacylation	0.031	0.047
	Cytosolic tRNA aminoacylation	0.035	0.047

*p*-values were adjusted by the Benjamini and Hochberg step-up procedure.

## Data Availability

Data used in the preparation of this article were obtained from the Parkinson’s Progression Markers Initiative (PPMI) database (www.ppmi-info.org/data (accessed on 10 November 2020)). For up-to-date information on the study, visit www.ppmi-info.org.
